# The gender difference in the effects of air pollution on the risk of spinal osteoarthritis in Chinese middle-aged and older adults: a prospective cohort study in China

**DOI:** 10.3389/fpubh.2025.1576204

**Published:** 2025-04-30

**Authors:** Jian Zhou, Guanghui Guo, Tao Liu, Zhenjun Zhu

**Affiliations:** ^1^Department of Orthopedics, Fourth Clinical College of Xinxiang Medical College, Xinxiang Central Hospital, Xinxiang, China; ^2^Department of Orthopedics, Hebei University of Engineering Affiliated Hospital, Handan, China

**Keywords:** spinal osteoarthritis, air pollution, cohort study, gender difference, China

## Abstract

**Objective:**

This study aimed to investigate whether exposure to multiple ambient air pollutants (PM_1_, PM_2.5_, PM_10_, O₃, and NO₂) elevates the risk of spinal osteoarthritis among middle-aged and older adults in China, and to further determine if there are gender-specific differences in vulnerability.

**Methods:**

A total of 7,663 participants aged 45 years and older, drawn from the China Health and Retirement Longitudinal Study (CHARLS), were followed from 2011 to 2020. Individuals free of spinal osteoarthritis at baseline were included. Annual mean concentrations of PM_1_, PM_2.5_, PM_10_, O₃, and NO₂ were extracted from the China High Air Pollutants (CHAP) dataset at a 1 km resolution (10 km for NO₂ in some years). Spinal osteoarthritis was identified via self-reported, physician-diagnosed cases involving the spine. Time-varying Cox proportional hazards regression models were used to evaluate hazard ratios (HR) and 95% confidence intervals (CI) per 10 μg/m^3^ increase in pollutant concentrations. All analyses accounted for demographic, socioeconomic, lifestyle, and spatial/seasonal factors, and explored potential effect modification by gender.

**Results:**

During the median 7-year (IQR: 4–9 years) follow-up, 1,556 participants newly reported spinal osteoarthritis. After adjusting for confounders, each 10 μg/m^3^ increment of PM_1_, PM_2.5_, PM_10_, and NO₂ was associated with a significant rise in the incidence of spinal osteoarthritis (13.8, 6.8, 5.1, and 17.4%, respectively), while O₃ showed a weaker and non-significant effect (1.1%). Notably, stratified analyses revealed that female participants exhibited pronounced vulnerability to PM_1_, PM_2.5_, PM_10_, and NO₂ exposures, whereas the associations in males were not statistically significant.

**Conclusion:**

This prospective study indicates that higher concentrations of particulate matter and traffic-related pollutants may contribute to an elevated risk of spinal osteoarthritis, particularly among women. These findings underscore the importance of incorporating musculoskeletal health into air quality management and highlight the value of targeted interventions—such as reducing ambient pollution and monitoring high-risk groups—to mitigate the burden of spinal osteoarthritis in rapidly urbanizing areas.

## Introduction

Osteoarthritis (OA) is a leading contributor to musculoskeletal disability worldwide, with an increasing prevalence driven in part by population aging and lifestyle changes (1, 2). Among its various forms, spinal OA is particularly concerning due to chronic back pain and disability, which can significantly impair quality of life in middle-aged and older adults (3). In China, the burden of OA has escalated alongside rapid socio-economic development, prompting a need to clarify its etiological risk factors and identify potential avenues for prevention (4, 5).

Air pollution has emerged as an important environmental determinant of health, implicated in cardiovascular, respiratory, and metabolic disorders (6). Accumulating evidence suggests that exposure to air pollutants such as particulate matter (PM) may also play a role in the onset or progression of joint diseases (7). For instance, a large cohort study in China linked prolonged PM_2.5_ exposure to accelerated knee OA progression, underlining a possible pro-inflammatory and oxidative stress-mediated mechanism (8–10). Although investigations have begun to elucidate the relationship between air pollution and peripheral joint OA, fewer studies have addressed spinal OA, despite its substantial prevalence and impact on functional independence (1, 11).

Gender differences in OA incidence, disease severity, and risk factor profiles have been reported, with women generally exhibiting higher susceptibility and more severe structural changes (12, 13). Nonetheless, whether these gender disparities extend to air pollution-related spinal OA remains unclear. Biological factors such as hormonal regulation and differential immune responses could modulate inflammatory processes in joint tissues, potentially altering susceptibility to environmental stressors (14–16). Moreover, behavioral and occupational exposures may differ by gender, further modifying the relationship between air pollutants and spinal OA risk (17, 18).

Given the paucity of research on how air pollution influences spinal OA in Asian populations—and particularly how this association may vary by gender—we conducted a prospective cohort study using nationally representative data from middle-aged and older Chinese adults. By capturing longitudinal exposure to multiple air pollutants (PM_1_, PM_2.5_, PM_10_, O₃, and NO₂), along with detailed demographic and health information, we aimed to determine the risk of spinal OA associated with ambient air pollution exposure, and assess potential differences in vulnerability between men and women. Our findings seek to inform targeted public health interventions that could mitigate OA-related disability in rapidly urbanizing settings.

## Methodology

### Study population and procedures

This investigation drew upon data from the China Health and Retirement Longitudinal Study (CHARLS), a nationally representative cohort administered by Peking University’s National School for Development (19). Initiated in 2011, CHARLS collects extensive information on health, socioeconomic background, and demographics from individuals aged 45 years and older across the country through multistage, stratified, and clustered household interviews. Follow-up surveys were conducted in 2013, 2015, 2018, and 2020 to capture updates in socio-demographic variables and health assessments. Ethical approval was granted by Peking University’s Biomedical Ethics Review Committee (IRB00001052-11015), and all participants provided informed consent.

**Figure 1:**
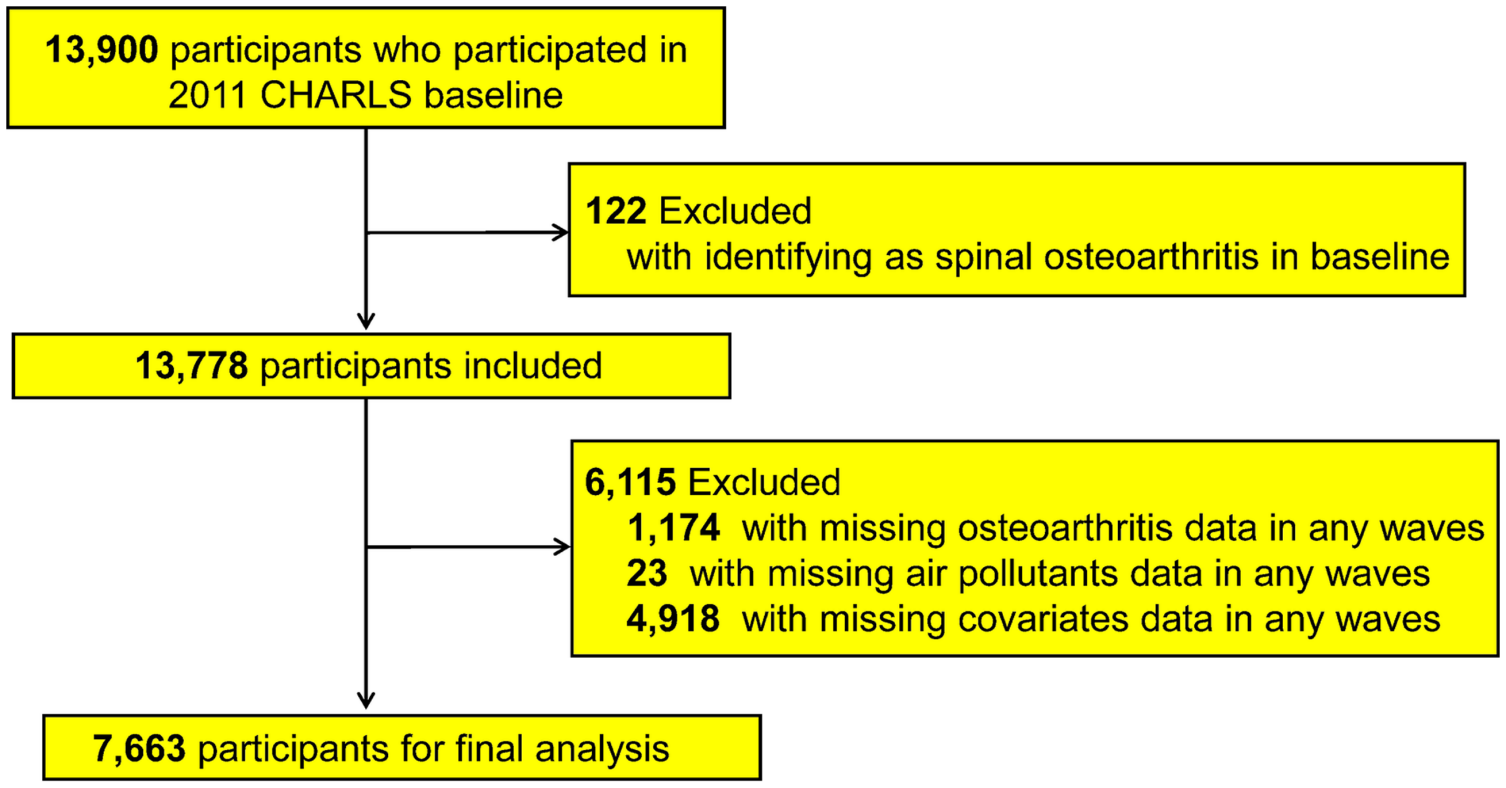
The flowchart of the selection process.

**Figure 2:**
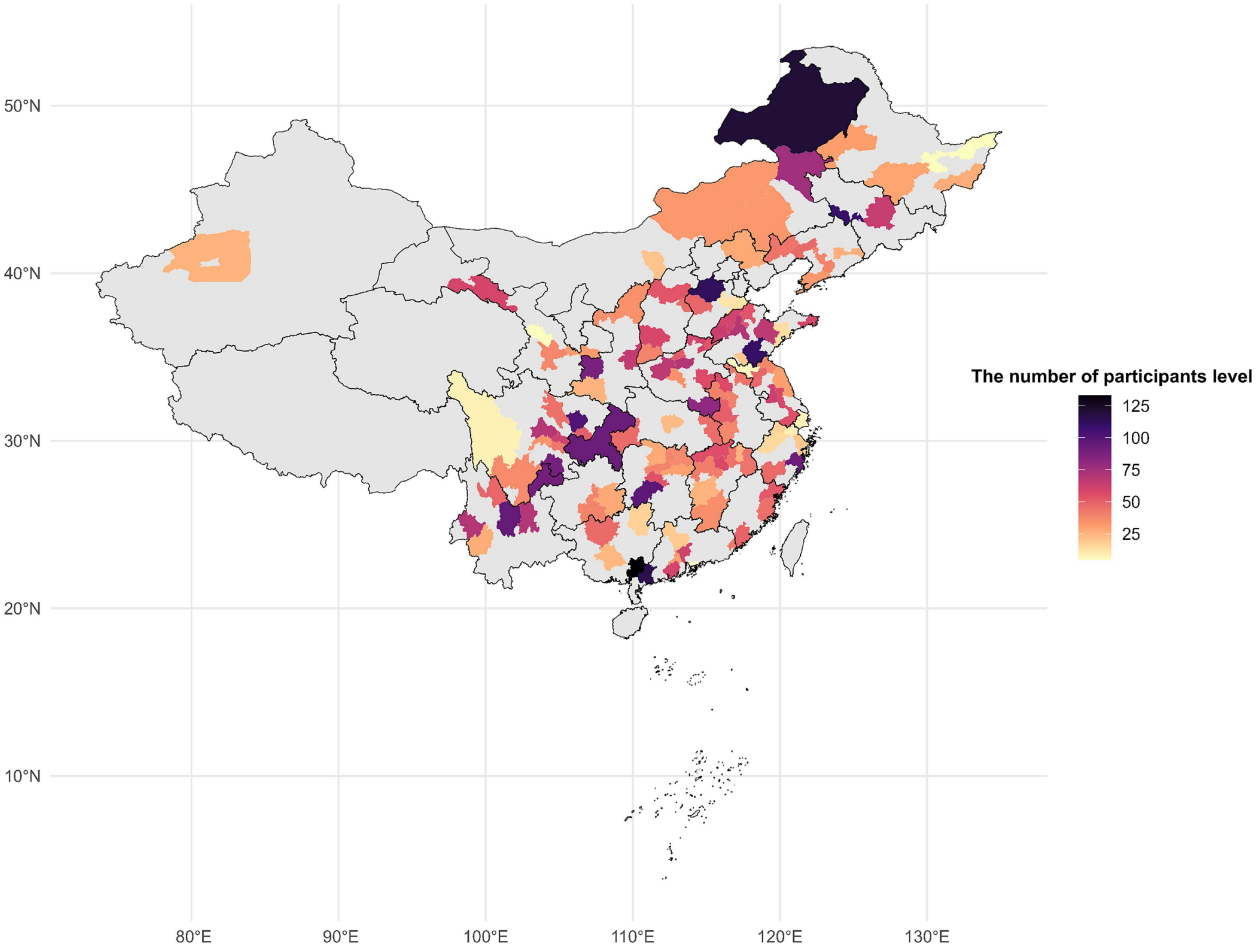
The distribution of the study participants for the study of the gender difference in the effects of air pollution on the risk of spinal osteoarthritis in Chinese middle-aged and older adults.

For the current study, participants were monitored from 2011 through 2020 using their unique CHARLS IDs. Out of the 13,900 respondents enrolled at baseline in 2011, we excluded 122 individuals who already had a recorded diagnosis of spinal osteoarthritis (or spinal-type osteoarthritis) at baseline, 1,174 missing spinal osteoarthritis data in any survey wave, 23 participants lacking data on air pollutant exposures, and 4,918 with insufficient covariate information. After exclusions, the 7,663 remaining analytic participants consisted of baseline osteoarthritis-free subjects with complete exposure and covariate data across the required waves. To address missing covariate information, the Last Observation Carried Forward (LOCF) method was used when appropriate (20). A flowchart of this selection process is presented in Figure 1. Figure 2 shows the distribution of the study participants in China.

### Exposure assessment: PM_1_, PM_2.5_, PM_10_, O_3_, and NO_2_

Annual, high-resolution concentrations of PM_1_, PM_2.5_, PM_10_, O_3_, and NO_2_ were retrieved from the China High Air Pollutants (CHAP) dataset. This resource provides year-round average pollutant concentrations (μg/m^3^) at a 1 km resolution for PM_1_, PM_2.5_, PM_10_, and O_3_ from January 2010 to December 2020, with NO_2_ values available at a 1 km or 10 km resolution. Based on geocoded community-level residence details from the CHARLS PSU dataset, each participant was assigned the previous calendar year’s mean pollutant exposure corresponding to their residential location, thus considering the lag effect of air pollution. For instance, the 2010 annual average concentration was linked to participants’ 2011 data in CHARLS.

### Outcome assessment: spinal osteoarthritis

Spinal osteoarthritis diagnoses were identified through self-reported physician-diagnosed osteoarthritis or rheumatism involving the spine. At every follow-up, participants were asked whether a healthcare professional had ever informed them that they had osteoarthritis or a degenerative joint condition in their back or spine. Additional questions captured the approximate timing of the condition’s onset. New-onset spinal osteoarthritis was defined as any new self-report of spinal osteoarthritis in a wave, provided the participant had no such report in prior waves. The first mention of spinal osteoarthritis served as the event of interest. Individuals who did not report spinal osteoarthritis, died, or were lost to follow-up were censored at their final available wave.

### Covariates

Covariates were chosen based on established risk factors for osteoarthritis and recognized confounders in air pollution research (16). These variables encompassed demographic aspects (age groups <70, 70–80, ≥80; gender: male/female; educational level: illiterate, primary or below, middle/high school, technical school or higher; urban/rural residence; and marital status: married/unmarried), socioeconomic measures (medical insurance type, income quintile, employment status), as well as lifestyle and sleep patterns (smoking behavior: current/former/never; alcohol status: current drinker/non-drinker; sleep duration: <7 h, 7–9 h, or >9 h). Adjustments for seasonal and spatial factors were incorporated using natural cubic splines of latitude and longitude, along with calendar year, month, and city. Whenever possible, these covariates were treated as time-varying, linking each participant’s reporting in a given wave to the pollutant exposure of the preceding year. Missing data were handled through the LOCF method, and certain stable demographic characteristics were imputed from baseline or inferred when consistent over time.

### Statistical analysis

Baseline comparisons were made between participants who developed spinal osteoarthritis during follow-up and those who did not, applying the Kolmogorov–Smirnov test to check variable distributions. We then utilized time-varying Cox proportional hazards regression, with survey waves as the time scale, to quantify hazard ratios (HR) and 95% confidence intervals (CI) associated with each 10 μg/m^3^ increment in annual mean air pollutant concentration. Four sequential models were implemented: Model 1 (unadjusted), Model 2 (adjusting for age and gender), Model 3 (further adjusting for socioeconomic status and seasonal/spatial factors), and Model 4 (additionally incorporating lifestyle and sleep variables). In addition, potential modifications by gender were evaluated using interaction terms (*Wald* test), and subgroup analyses stratified by gender were performed to explore effect modification. Furthermore, multiple pollutant models (both two- and three-pollutant) were employed based on Model 4, which accounts for all confounding factors. Finally, the sensitivity analysis was conducted using pollutant data with a two-year lag. Analyses were conducted in Stata 16.0 (StataCorp, College Station, TX) and R (version 4.3.2), with statistical significance determined at a two-sided *p*-value <0.05.

## Results

Table 1 illustrates the baseline characteristics of the 7,663 participants stratified by spinal osteoarthritis status (no spinal osteoarthritis vs. spinal osteoarthritis). Of these individuals, 6,107 (79.7%) did not report spinal osteoarthritis, whereas 1,556 (20.3%) had spinal osteoarthritis. Compared to participants free of spinal osteoarthritis, those with spinal osteoarthritis were more likely to be female, older, hold a technical school or higher education, be unmarried, live in urban areas, be former smokers, abstain from alcohol, have UEMI/URRMI/URMI medical insurance, fall into the lowest income category, be unemployed, and engage in less physical activity (Table 1).

**Table 1:** Baseline characteristics of 7,663 participants stratified by spinal osteoarthritis.

Variables	Total (*n* = 7,663)	Non-hypertension (*n* = 6,107)	Hypertension (*n* = 1,556)
Gender			
Female	4,060 (53.0)	3,237 (53.0)	823 (52.9)
Male	3,603 (47.0)	2,870 (47.0)	733 (47.1)
Age groups (years)			
<70	6,661 (86.9)	5,343 (87.5)	1,318 (84.7)
70–80	820 (10.7)	629 (10.3)	191 (12.3)
≥80	182 (2.4)	135 (2.2)	47 (3.0)
Educational level			
Illiterate	1,954 (25.5)	1,592 (26.1)	362 (23.3)
Primary school and below	3,020 (39.4)	2,470 (40.5)	550 (35.4)
Middle school and high school	2,138 (27.9)	1,752 (28.7)	386 (24.8)
Technical school and above	551 (7.2)	293 (4.8)	258 (16.6)
Marriage			
Unmarried	862 (11.3)	672 (11.0)	190 (12.2)
Married	6,801 (88.7)	5,435 (89.0)	1,366 (87.8)
Residence			
Urban	2,720 (35.5)	2,162 (35.4)	558 (35.9)
Rural	4,943 (64.5)	3,945 (64.6)	998 (64.1)
Smoke			
Never smoking	4,612 (60.2)	3,673 (60.2)	939 (60.3)
Former smoking	613 (8.0)	470 (7.7)	143 (9.2)
Still smoking	2,438 (31.8)	1,964 (32.2)	474 (30.5)
Alcohol consumption			
Not drinking	5,080 (66.3)	4,040 (66.1)	1,040 (66.8)
Current drinking	2,583 (33.7)	2,067 (33.9)	516 (33.2)
Medical insurance			
NMI^*^	513 (6.7)	415 (6.8)	98 (6.3)
UEMI^*^	590 (7.7)	470 (7.7)	120 (7.7)
URRMI^*^	92 (1.2)	67 (1.1)	25 (1.6)
URMI^*^	299 (3.9)	232 (3.8)	67 (4.3)
NCMI^*^	5,962 (77.8)	4,758 (77.9)	1,204 (77.4)
Others	207 (2.7)	165 (2.7)	42 (2.7)
Income group			
Quartile 1: 0 to 2,880¥^*^	6,263 (81.8)	4,976 (81.4)	1,287 (82.7)
Quartile 2: 2,880¥ to 11,000¥^*^	514 (6.7)	417 (6.8)	97 (6.2)
Quartile 3: 11,000¥ to 21,900¥^*^	450 (5.9)	371 (6.1)	79 (5.1)
Quartile 4: 21,900¥ to 41,000¥^*^	349 (4.6)	279 (4.6)	70 (4.5)
Quartile 5: greater than 41,000¥^*^	87 (1.1)	64 (1.0)	23 (1.5)
Employment status			
Unemployed	2,515 (32.8)	1,973 (32.3)	542 (34.8)
Agricultural job	4,115 (53.7)	3,312 (54.2)	803 (51.6)
Non-agricultural job	1,033 (13.5)	822 (13.5)	211 (13.6)
Sleep duration			
Short^*^	3,746 (48.9)	2,988 (48.9)	758 (48.7)
Normal^*^	3,456 (45.1)	2,763 (45.3)	693 (44.6)
Long^*^	461 (6.0)	356 (5.8)	105 (6.8)
Physical activity			
Physically inactive	1,280 (16.7)	742 (12.2)	538 (34.6)
Physically active	6,383 (83.3)	5,365 (87.8)	1,018 (65.4)
Air pollutants			
PM_1_^*^	25.5 (18.1, 33.8)	25.7 (19.0, 34.2)	25.6 (18.8, 33.7)
PM_2.5_^*^	44.5 (32.2, 59.6)	45.2 (33.0, 61.2)	45.1 (32.5, 60.2)
PM_10_^*^	77.0 (54.0, 104.0)	78.7 (55.2, 106.3)	79.2 (56.0, 105.1)
O_3_^*^	88.5 (82.0, 97.0)	86.8 (80.9, 95.6)	87.1 (82.1, 97.4)
NO_2_^*^	25.5 (19.1, 33.5)	26.0 (19.6, 33.6)	26.3 (19.7, 33.9)

^*^ Non-medical insurance (NMI); Non-medical insurance (NMI); Urban employee medical insurance (UEMI); Urban and rural resident medical insurance (URRMI); Urban resident medical insurance (URMI); New cooperative medical insurance (NCMI); Quartile 1, personal annual income of 0 to 2,880¥; Quartile 2, personal annual income of 2,880¥ to 11,000¥; Quartile 3, personal annual income of 11,000¥ to 21,900¥; Quartile 4, personal annual income of 21,900¥ to 41,000¥; Quartile 5, personal annual income of greater than 41,000¥; Sleep duration, short (nighttime sleep duration < 7 h), normal (nighttime sleep duration 7–9 h), long (nighttime sleep duration > 9 h); Physical activity (physical inactivity and physically active); The data related to air pollutants represent the median (25th percentile, 75th percentile) of the annual average concentrations (μg/m^3^).

**Figure 3:**
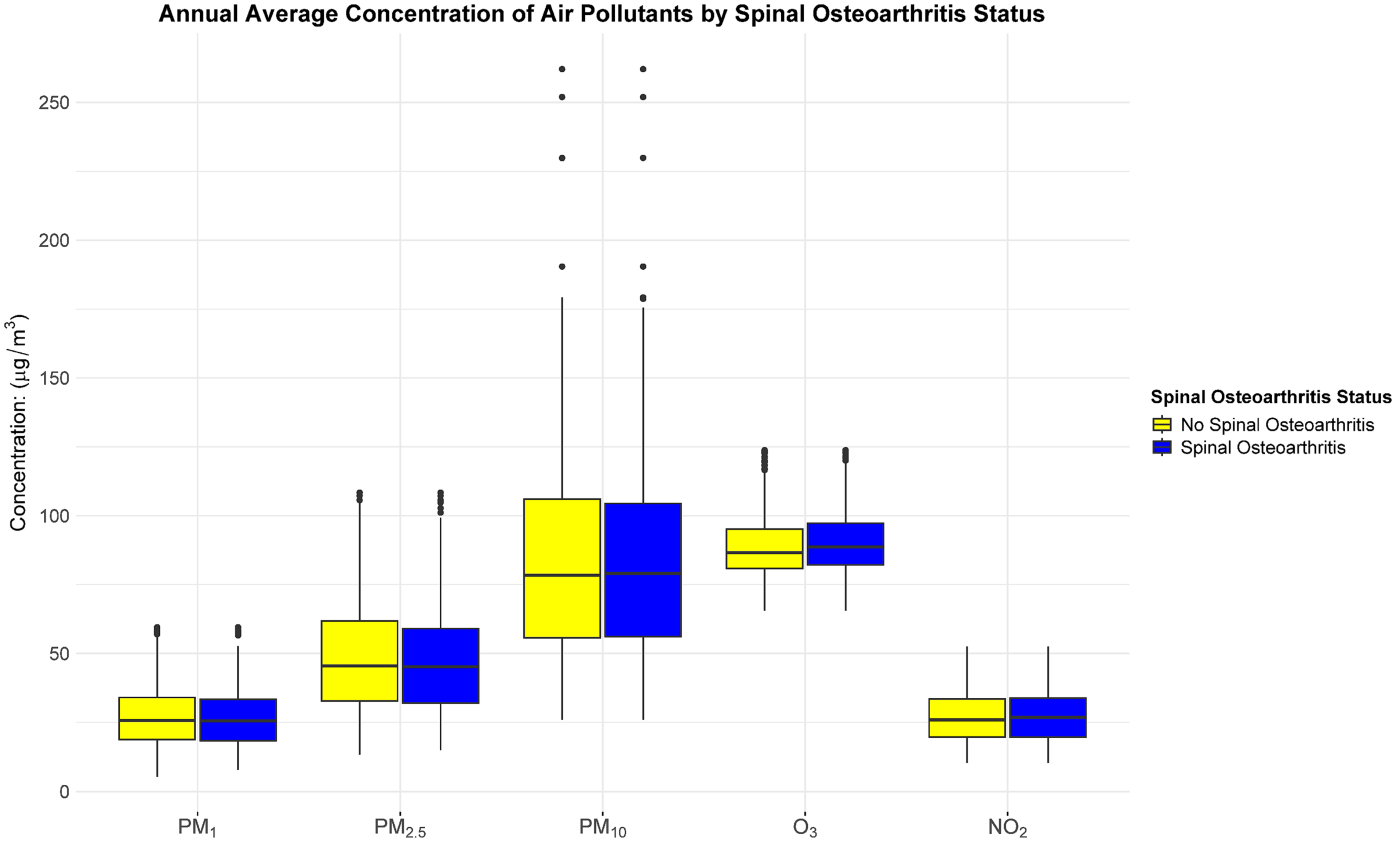
Annual average concentration of air pollutants by spinal osteoarthritis status.

**Figure 4:**
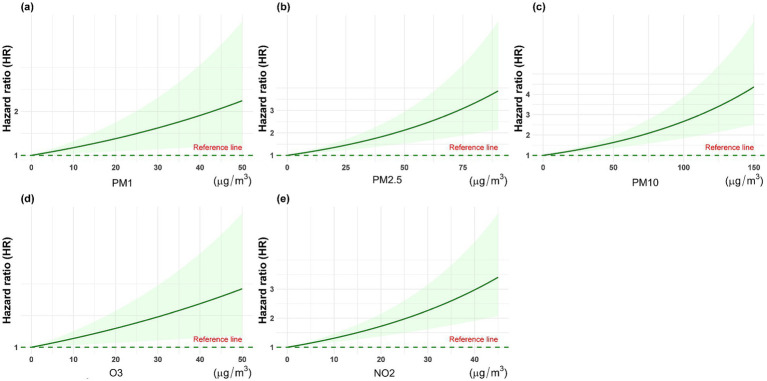
The restricted cubic spline plots for the association between the annual concentrations of each air pollutant and the risk of spinal osteoarthritis. **(a)** PM_1_, **(b)** PM_2.5_, **(c)** PM_10_, **(d)** O_3_, and **(e)** NO_2_.

Based on the Kolmogorov–Smirnov test, the annual average concentrations of the five pollutants (PM_1_, PM_2.5_, PM_10_, O_3_, and NO_2_) deviated from a normal distribution (*p* < 0.05). Figure 3 shows the annual average concentration of air pollutants by spinal osteoarthritis status. Hence, they are presented as median and interquartile range. Following a median follow-up of 7 years (IQR: 4–9 years), a total of 1,556 participants were classified as having spinal osteoarthritis. After adjusting for all potential confounders (Model IV), each 10 μg/m^3^ increment in annual mean exposure to PM_1_, PM2.5, PM_10_, O_3_, and NO_2_ was associated with a 13.8% (HR = 1.138, 95% CI 1.085–1.195), 6.8% (HR = 1.068, 95% CI 1.043–1.093), 5.1% (HR = 1.051, 95% CI 1.036–1.067), 1.1% (HR = 1.011, 95% CI 0.983–1.041), and 17.4% (HR = 1.174, 95% CI 1.113–1.239) higher risk of developing spinal osteoarthritis, respectively (Table 2). Figure 4 presents the restricted cubic spline plots for the association between the annual concentrations of each air pollutant and the risk of spinal osteoarthritis. The sensitivity analysis for two-years lag effect shows that there was no significant difference with the results of main analysis (Appendix).

**Table 2:** The associations between air pollutants exposure (10 μg/m^3^) and the risk of spinal osteoarthritis in 7,663 participants: the results of time-varying Cox regression analysis.

Variables	Model I	Model II	Model III	Model IV
	HR (95%CI)	HR (95%CI)	HR (95%CI)	HR (95%CI)
No spinal osteoarthritis	1.000	1.000	1.000	1.000
PM_1_/spinal osteoarthritis	1.009 (0.997–1.021)	1.022 (1.010–1.034)	1.145 (1.091–1.201)	1.138 (1.085–1.195)
PM_2.5_/spinal osteoarthritis	1.006 (1.000–1.012)	1.017 (1.010–1.024)	1.072 (1.048–1.097)	1.068 (1.043–1.093)
PM_10_/spinal osteoarthritis	1.011 (1.007–1.015)	1.019 (1.015–1.024)	1.054 (1.039–1.070)	1.051 (1.036–1.067)
O_3_/spinal osteoarthritis	1.033 (1.020–1.046)	1.028 (1.015–1.041)	1.014 (0.985–1.043)	1.011 (0.983–1.041)
NO_2_/spinal osteoarthritis	1.058 (1.044–1.073)	1.075 (1.061–1.090)	1.188 (1.126–1.253)	1.174 (1.113–1.239)

Model I, no covariates were adjusted; Model II: adjusted for age group and gender; Model III: additionally adjusted for socioeconomic status (SES) factors (educational level, residence (urban or rural), marital status, medical insurance, income, employment status), season and spatial autocorrelation; Model IV: as Model III adjusted for behavioral factors (smoking status, alcohol consumption, sleep duration and physical activity).

Table 3 displays the outcomes for models incorporating multiple pollutants. Under single-pollutant conditions, O₃ showed no discernible link to spinal osteoarthritis, and this pattern persisted even after accounting for additional pollutants. In contrast, the associations of the remaining pollutants with spinal osteoarthritis held steady when O₃ was included, each with a slightly increased effect size. Once NO₂ was introduced, the relationships involving PM₁ and PM_2.5_ ceased to be statistically significant, whereas PM₁₀ exhibited the opposite trend. Moreover, NO₂‘s connection to frailty remained largely independent of the other pollutants.

**Table 3:** The associations between air pollutants exposure (10 μg/m^3^) and the risk of spinal osteoarthritis in 7,663 participants: the models with single and multiple pollutants.

Air pollutants	Single model	+ O₃	+ NO₂	+ O₃ + NO₂	+ PM₁	+ PM_2.5_	+ PM₁₀	+ PM₁ + NO₂	+ PM_2.5_ + NO₂	+ PM₁₀ + NO₂
PM₁	1.138 (1.085–1.195)	1.145 (1.091–1.202)	1.040 (0.980–1.105)	1.035 (0.970–1.100)	–	–	–	–	–	–
PM_2.5_	1.068 (1.043–1.093)	1.073 (1.047–1.100)	1.025 (0.975–1.073)	1.020 (0.970–1.068)	–	–	–	–	–	–
PM₁₀	1.051 (1.036–1.067)	1.057 (1.042–1.073)	1.044 (1.027–1.062)	1.040 (1.023–1.059)	–	–	–	–	–	–
O₃	1.011 (0.983–1.041)	–	1.008 (0.978–1.039)	–	1.010 (0.980–1.041)	1.012 (0.982–1.043)	1.015 (0.985–1.046)	1.005 (0.975–1.038)	1.007 (0.977–1.040)	1.008 (0.978–1.042)
NO₂	1.174 (1.113–1.239)	1.180 (1.118–1.246)	–	–	1.165 (1.105–1.232)	1.160 (1.098–1.228)	1.155 (1.095–1.225)	–	–	–

**Figure 5:**
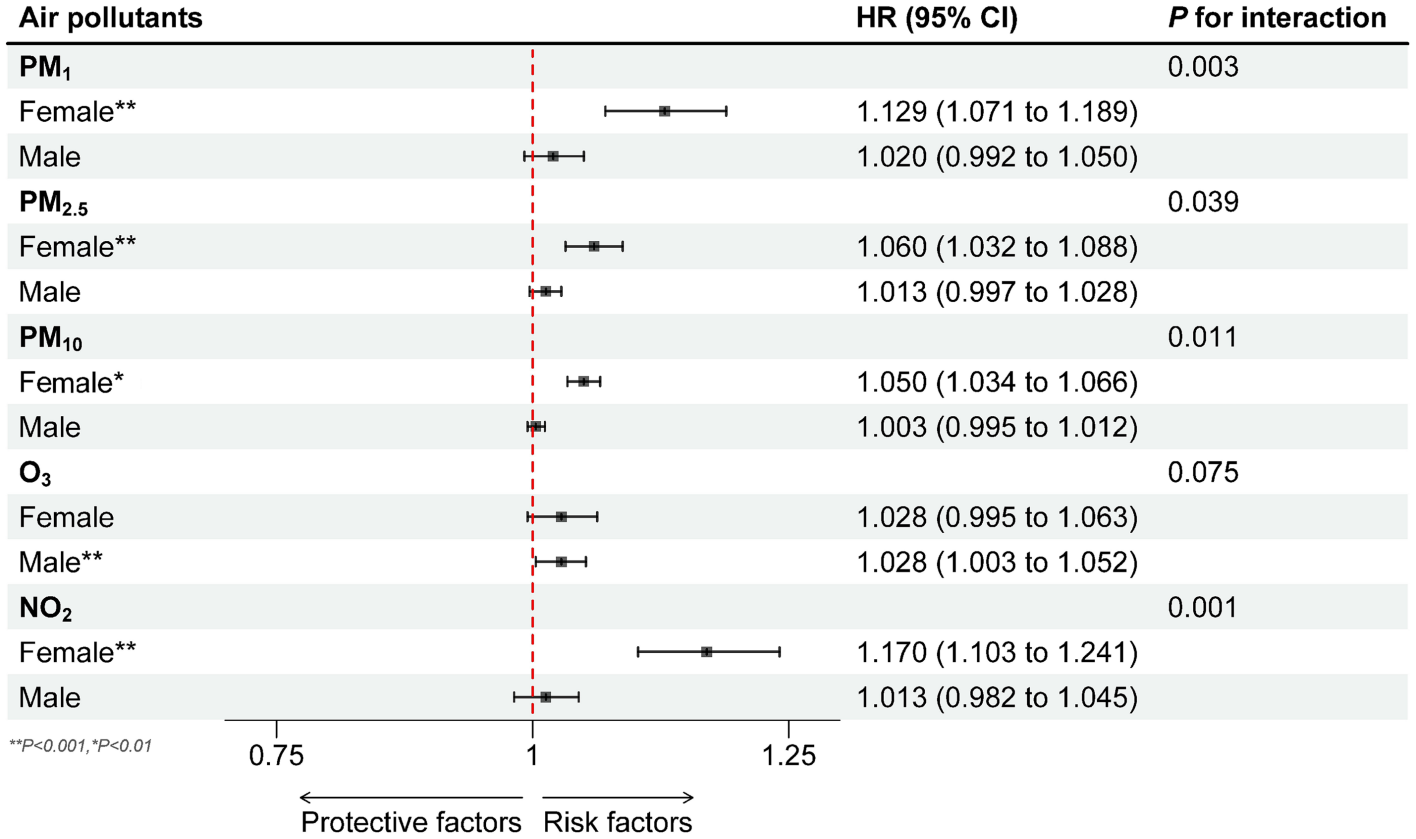
Subgroup analysis of the relationship of PM_1_, PM_2.5_, PM_10,_ O_3_ and NO_2_ exposure (10 μg/m^3^) with the risk of spinal osteoarthritis in different physical activity among 7,663 participants: the results of time-varying Cox regression analysis. Separate analyses were performed for each one of the pollutants and each analysis was adjusted for age group, gender, socioeconomic status (SES) factors (educational level, residence (urban or rural), marital status, medical insurance, income, employment status), season and spatial autocorrelation, behavioral factors (smoking status, alcohol consumption, sleep duration and physical activity).

The interaction tests for PM_1_, PM_2.5_, PM_10_ and NO_2_ with gender were significant (all *P* for interaction less than 0.05), and only the O_3_ was not significant (*P* for interaction: 0.19). Further subgroup analysis showed that for male participants, the association between PM_1_, PM_2.5_, PM_10_ and NO_2_ exposure and the spinal osteoarthritis risk was not significant (*p* > 0.05). In contrast, for female subgroup, PM_1_, PM_2.5_, PM_10_ and NO_2_ exposure significantly increased the risk of spinal osteoarthritis by 12.9% (HR = 1.129, 95% CI 1.071–1.189), 6.0% (HR = 1.060, 95% CI 1.032–1.088), 5.0% (HR = 1.050, 95% CI 1.034–1.066), 17.0% (HR = 1.170, 95% CI 1.103–1.241) (Figure 5).

## Discussion

Our prospective study of 7,663 community-dwelling Chinese adults has demonstrated a positive relationship between elevated ambient concentrations of PM_1_, PM_2.5_, PM_10_, and NO₂ and increased susceptibility to spinal osteoarthritis over a median follow-up of 4 years. In contrast, O₃ exposure displayed a weaker correlation with spinal OA onset. Notably, participants with spinal osteoarthritis were more likely to be older, female, living in urban areas, and less physically active, suggesting that both demographic and behavioral factors may synergistically exacerbate the detrimental effects of airborne pollutants. These findings offer a nuanced perspective on the environmental determinants of musculoskeletal health, extending beyond the conventional emphasis on cardiovascular and respiratory diseases.

Recent epidemiological and experimental evidence supports the plausibility of pollutant-induced degenerative changes in joint tissues. In particular, mounting data suggest that fine particulate matter and traffic-related emissions may provoke systemic oxidative stress, chronic low-grade inflammation, and impaired microvascular function (21, 22). Our results are congruent with those of one study (3), who reported that increased exposure to PM_2.5_ and PM_10_ was associated with a heightened prevalence of osteoarthritis among Chinese middle-aged and older adults. Additionally, another study identified PM_2.5_ as a potential risk factor for rapid knee OA progression, proposing that deposited particulate matter within the joint environment could contribute to cartilage breakdown (23, 24). Although fewer studies have focused specifically on spinal OA, our findings highlight its potential susceptibility to similar inflammatory pathways. Future mechanistic investigations—potentially using imaging modalities or biomarkers of inflammation—could further elucidate how airborne toxins drive molecular changes in intervertebral discs and facet joints (25, 26).

A key observation in our analysis is the notable gender disparity, with female participants appearing more vulnerable to pollution-related joint deterioration. This aligns with previous studies based on the biological and behavioral and socio-environmental perspectives (27, 28). From a biological standpoint, hormonal regulation and immunological factors in women may contribute significantly to this heightened susceptibility. Estrogen, for instance, has been shown to exert protective effects on cartilage by modulating inflammatory cytokines (e.g., IL-1β, TNF-*α*) and promoting collagen synthesis. After menopause, the decline in estrogen levels may create a more pro-inflammatory and oxidative environment, potentially exacerbating the adverse impact of environmental toxins on joint structures (29–31). Furthermore, emerging research suggests that women could exhibit distinct immunological responses to particulate matter, including greater expression of reactive oxygen species and inflammatory mediators, which could accelerate cartilage and subchondral bone damage (32, 33).

In addition to these biological factors, behavioral and socio-environmental dimensions may also contribute to women’s higher susceptibility. In many cultures, older women often assume more indoor responsibilities, such as cooking, which can increase exposure to household air pollutants (e.g., from biomass fuels or inadequate ventilation) (31). Occupational factors may also play a role: women in certain urban service or informal jobs may face prolonged outdoor or semi-enclosed exposures to traffic-related pollutants, especially if protective measures are lacking. Moreover, caregiving and domestic duties can limit leisure-time physical activity, reducing muscular support around the spine and potentially heightening the detrimental effects of pollution on weight-bearing structures (32, 34).

Despite the robustness of our longitudinal design and adjustment for a wide array of demographic, socioeconomic, and lifestyle covariates, several limitations warrant discussion. First, our reliance on city-level pollutant data might underestimate inter-individual variation in exposure, as local traffic patterns or building environments could produce marked differences in actual inhaled doses (35). Efforts to incorporate personal monitoring devices or satellite-based micro-scale estimations may refine future risk assessments (36, 37). Second, the identification of spinal osteoarthritis was based on self-reported physician diagnoses rather than imaging or clinical examination, raising concerns about potential misclassification—particularly in early or asymptomatic cases (16, 38). Third, while we included known confounders, unmeasured factors such as dietary habits, vitamin D status, or coexisting metabolic diseases may also influence osteoarthritis outcomes. Lastly, the omission of multi-pollutant modeling means we have a limited view of interactive or additive effects among different pollutants (35, 39, 40).

From a public health perspective, our findings underscore the urgent need for policies and interventions aimed at reducing ambient concentrations of PM and NO₂ in rapidly urbanizing regions. The link between these pollutants and spinal OA risk represents an underrecognized domain of morbidity that may add to healthcare burdens, given the chronic and disabling nature of back pain. In particular, more focused interventions on traffic-related pollutants could involve the implementation of low-emission zones, stricter tailpipe standards, catalytic converter mandates, and congestion-pricing policies to curb vehicular emissions. Additionally, expanding community-based air quality monitoring networks in heavily polluted districts would allow for more precise identification of high-risk areas and facilitate timely public health alerts. Such efforts should be accompanied by improved public transit systems and the strategic development of green spaces, which not only reduce pollution but also encourage physical activity (41, 42). Furthermore, heightened surveillance and targeted prevention initiatives—especially for older women—could mitigate pollution’s detrimental impact on skeletal health. These programs might incorporate routine musculoskeletal screenings in clinics situated in high-emission neighborhoods, along with educational campaigns to increase awareness of air pollution’s potential role in joint degeneration. As many older women may have additional exposures from household cooking or occupational settings, multi-sector collaboration is crucial to minimize total pollutant loads. Investigating the joint influences of occupational exposures, household air pollution, and broader environmental factors remains critical in developing effective, context-specific strategies to preserve spinal health in aging societies (43, 44). Future studies should also incorporate advanced clinical imaging techniques (e.g., MRI, CT, or cartilage compositional imaging) and molecular profiling (e.g., inflammatory biomarkers, epigenetic analyses) to elucidate the precise pathophysiological mechanisms by which air pollutants may accelerate degenerative changes in the spine. Moreover, continued exploration of these specific mechanisms using more diverse data sources—such as wearable monitoring devices, high-resolution regional pollution metrics, and longitudinal biobanks—will be essential to further validate and expand upon our current research findings, ultimately guiding more targeted and effective public health interventions.

## Conclusion

In this nationwide longitudinal analysis, exposure to elevated levels of PM_1_, PM_2.5_, PM_10_, and NO₂ was consistently linked to a higher incidence of spinal osteoarthritis, while O₃ demonstrated a comparatively weaker association. Our data also indicate that women may be especially sensitive to the harmful effects of air pollution on spinal structures. These observations contribute to a growing body of literature suggesting that environmental pollutants, long implicated in cardiopulmonary conditions, also pose significant risks to musculoskeletal integrity. By pinpointing high-risk populations and key pollutant sources, policymakers and clinicians can collaborate on interventions—ranging from emission controls to health education campaigns—that may reduce the overall burden of osteoarthritis and promote healthier aging. Further research integrating advanced exposure assessment, clinical imaging, and molecular profiling is warranted to fully characterize the mechanisms linking air pollutants to spinal joint degeneration.

## Data Availability

The original contributions presented in the study are included in the article/supplementary material, further inquiries can be directed to the corresponding author.

## Appendix

**Table A1:** The sensitivity analysis for the associations between air pollutants exposure (10 μg/m^3^) and the risk of spinal osteoarthritis in 7,663 participants: the results of two-years lag effect for air pollution.

Variables	Model I	Model II	Model III	Model IV
	HR (95%CI)	HR (95%CI)	HR (95%CI)	HR (95%CI)
No spinal osteoarthritis	1.000	1.000	1.000	1.000
PM_1_/spinal osteoarthritis	1.015 (1.003–1.027)	1.029 (1.017–1.041)	1.137 (1.083–1.193)	1.130 (1.078–1.188)
PM_2.5_/spinal osteoarthritis	1.013 (1.005–1.019)	1.023 (1.016–1.030)	1.065 (1.040–1.091)	1.061 (1.037–1.086)
PM_10_/spinal osteoarthritis	1.015 (1.010–1.019)	1.026 (1.022–1.031)	1.050 (1.035–1.067)	1.047 (1.032–1.063)
O_3_/spinal osteoarthritis	1.027 (1.014–1.040)	1.022 (1.009–1.035)	1.018 (0.989–1.046)	1.006 (0.978–1.037)
NO_2_/spinal osteoarthritis	1.052 (1.038–1.067)	1.069 (1.055–1.084)	1.177 (1.117–1.243)	1.164 (1.104–1.229)

Model I, no covariates were adjusted; Model II: adjusted for age group and gender; Model III: additionally adjusted for socioeconomic status (SES) factors (educational level, residence (urban or rural), marital status, medical insurance, income, employment status), season and spatial autocorrelation; Model IV: as Model III adjusted for behavioral factors (smoking status, alcohol consumption, sleep duration and physical activity).
